# The Effect of Perceived Threat Avoidability of COVID-19 on Coping Strategies and Psychic Anxiety Among Chinese College Students in the Early Stage of COVID-19 Pandemic

**DOI:** 10.3389/fpsyt.2022.854698

**Published:** 2022-03-30

**Authors:** Jinnan Wu, Yelianghui Zheng, Shankuo Xiong, Wenpei Zhang, Shanshan Guo

**Affiliations:** Department of Business Administration, School of Business, Anhui University of Technology, Ma’anshan, China

**Keywords:** anxiety, cognition, coping strategy, COVID-19, mental health

## Abstract

**Background:**

The novel coronavirus disease 2019 (COVID-19) outbreak has seriously threatened the mental health of college students. This study intended to invest whether perceived threat avoidability of COVID-19 relates to psychic anxiety among college students during the early stage of the COVID-19 pandemic, as well as the mediating roles of COVID-19-specific wishful thinking and COVID-19-specific protective behaviors in this relationship.

**Methods:**

A cross-sectional study was conducted in China, using a random sampling method (February 6–25, 2020). Self-reported questionnaires were conducted online included the Perceived Threat Avoidability of COVID-19 Scale, COVID-19-specific Wishful Thinking Scale, COVID-19-specific Protective Behaviors Scale, and the Hamilton Psychogenic Anxiety Scale. The data were analyzed using Structural equation modeling and Bootstrapping procedure.

**Results:**

A total of 2922 samples were collected in this study. Perceived threat avoidability of COVID-19 is negatively related to psychic anxiety (β = −0.158, *p*< 0.001), and both COVID-19-specific wishful thinking (β = −0.006, *p* = 0.029, 95% CI: [−0.012, −0.001]) and protective behaviors (β = −0.029, *p*< 0.001, 95% CI: [−0.043, −0.018]) mediate this relationship. Also, COVID-19-specific wishful thinking is found to correlate with COVID-19-specific protective behaviors negatively (β = −0.112, *p* < 0.001).

**Conclusion:**

Perceived threat avoidability of COVID-19 contributes to psychic anxiety among college students. COVID-19-specific wishful thinking strategy plays a negative mediating role and increases the level of anxiety; COVID-19-specific protective behaviors strategy plays a positive mediating role and reduces the level of anxiety; meanwhile, wishful thinking also suppresses college students from adopting protective behaviors.

## Introduction

The novel coronavirus disease (COVID-19) pandemic, declared as a public health emergency of international concern by the World Health Organization (WHO) on 30 January 2020 (1), carries a global and acute threat to public health (2). In addition to the threat to physical health, the unpredicfpsyt-13-798480_SFTPility and outbreak of COVID-19, as well as the lack of social interaction caused by mandatory social distancing, seriously threatens the public’s mental health (e.g., fear and anxiety) (3). Moreover, constant COVID-19-related rumors on the Internet aggravate group panic and cause anxiety among different groups (4, 5). Compared with other periods of the COVID-19 pandemic, COVID-19 creates more unknowns to the general public and healthcare workers in the early stage of the COVID-19 pandemic, such as unknown virus sources, pathogenic mechanisms, effective treatments, and preventive measures. The uncertainties brought by these unknowns make the public more susceptible to anxiety in the early stage of the COVID-19 pandemic (6). Therefore, it is valuable for researchers and policy-makers to identify the predictors of public anxiety toward the COVID-19 pandemic and reveal the underlying mechanism in the early stage of the COVID-19 pandemic.

Previous research has found that young adults (e.g., college students) are more sensitive to information about the pathogeny, infectiousness, cure rates and mortality of the severe acute respiratory syndrome (SARS), whose negative emotional responses such as anxiety and panic are more pronounced than other groups (7, 8). More specifically, Sun et al. (9) suggested that college students are more vulnerable to the psychological consequences of the COVID-19 pandemic. Several studies have shown that the COVID-19 pandemic leads to the deterioration of mental health conditions in college students, such as significantly high levels of depression (10) and the generation of anxiety (11). However, the mental health of college students has received much less attention than that of healthcare workers. In the early stage of the COVID-19 pandemic in China, the first country reporting the COVID-19 pandemic to the WHO, Chinese college students were not only exposed to the direct threat of unknown COVID-19, but also were required to adhere to strict home quarantine policies and receive distance learning. These changes in their lives and studies have damaged physical health, limited social interaction, reduced physical activities, and altered learning styles, leading to a significant increase in psychic anxiety symptoms among Chinese college students (12, 13). Further, in the early stage of the COVID-19 pandemic, Chinese college students’ anxiety levels were higher than the norm score of other adults, and non-medical college students had more severe anxiety than medical college students. Because non-medical college students have more uncertainties about the COVID-19 pandemic and feel more anxiety than medical college students who have a rich background of medical knowledge, and they need more psychological support in terms of cognitive and negative emotional interventions (14). In addition, the larger size of non-medical students makes the results of this study more widely beneficial. Hence, this research aims to narrow the gap in the existing literature by focusing on which factors relate to COVID-19-related psychic anxiety among non-medical college students in the early stage of the COVID-19 pandemic in China.

The existing studies on COVID-19-related anxiety of college students have well examined the prevalence and levels of anxiety, and the demographic and coping antecedents of anxiety. First, Tang et al. (13) found that the proportion of clinically elevated anxiety symptoms was 15.4% in a top university in China. Islam et al. (15) indicated that 18.1% of Bangladeshi college students suffered from severe anxiety disorders. Second, several studies have examined the relationship between demographic factors (e.g., sex, age, residence, nationality, parents’ social status, etc.) and COVID-19-related anxiety of college students (15–18). Third, studies on the effects of coping strategies and social media use on anxiety found that positive problem-focused and emotion-focused coping strategies were related to a low level of COVID-19-related anxiety among college students (19, 20). These three streams of research provide valuable insights into our understanding of college students’ anxiety during the COVID-19 pandemic. However, little is known about whether and how perceived threat avoidability of COVID-19, which is inherent in uncertainties of COVID-19 in the early stage of the pandemic in China, causes college students’ psychic anxiety.

Research based on the terror management theory, which understands the impact of COVID-19 threat on people, suggests that people respond differently to COVID-19 threat, and ineffective coping may produce psychological distress, emphasizing the important role of coping in COVID-19 and psychological distress (e.g., anxiety) (21, 22). Further, cognitive appraisal theory, which is used to explain stress appraisal and coping, can provide a theoretical basis for analyzing the specific processes by which individuals cope with COVID-19 in different ways (23). Cognitive appraisal theory states that negative emotions (e.g., psychic anxiety) are responses of individuals after a series of appraisals and coping with harmful environmental events (6, 24, 25). Individuals generally make a primary *threat appraisal* regarding the severity of the threat itself (i.e., perceived threat severity), followed by a secondary *coping appraisal* regarding their ability to cope (i.e., perceived threat avoidability in this study), and finally adopt *coping strategies* including emotion-focused coping (EFC) and problem-focused coping (PFC) (6, 23, 26). Confronted with the COVID-19 pandemic, a major unexpected harmful event, individuals widely have a high level of threat evaluation of COVID-19 and induce psychic anxiety (9, 24, 27). While few studies to date have examined the effect of coping appraisal (e.g., sense of control) on the COVID-19-induced psychic anxiety (28), the underlying mechanism for explaining this effect is underexplored. Further, previous studies have supported the association of positive PFC and EFC strategies with low levels of COVID-19-induced anxiety among college students during the COVID-19 pandemic (19, 20), and the prevention of mental health deterioration in college students by scientific coping methods (e.g., quarantine policy) (29), suggesting a possible beneficial role of coping strategies in coping appraisal and psychic anxiety. Chen and Liang (30) further confirmed that coping appraisal influences users’ behavioral intention through the mediations of PFC and EFC strategies. Thus, this study seeks to narrow the gaps in theories by examining the relationship between college students’ coping appraisal (perceived threat avoidability of COVID-19) and their psychic anxiety, as well as the mediation of EFC and PFC strategies toward COVID-19.

Among EFC strategies such as wishful thinking, expressing emotions, self-criticism, and social withdrawal (31), wishful thinking has been proven to be one of the most important coping strategies influencing individuals’ anxiety and behavioral responses (32–35). Therefore, this study focuses on wishful thinking and its relation to perceived threat avoidability and psychic anxiety. Further, Folkman and Lazarus (35) noticed that some forms of EFC strategies could affect PFC strategies. A recent study demonstrates a negative effect of wishful thinking on PFC when users face an information technology threat (36). Thus, this study further tests the relationship between wishful thinking and protective behaviors in the context of the COVID-19 pandemic.

The rest of this paper is organized as follows. Our hypotheses are described at first. Next, methodology and data analysis results are presented. Then, we conclude this paper by discussing the findings, theoretical and practical implications, limitations, and future directions.

## Hypotheses Development

### Perceived Threat Avoidability of COVID-19 and Psychic Anxiety

The basic assumption of cognitive appraisal theory is that emotion is an individual’s perceived beneficial or harmful response to environmental events and is a complex conceptualization of the appraisal process (6). Cognitive appraisal theory consists of two core concepts: appraisal and coping. The appraisal can be further divided into primary appraisal, in which individual evaluates whether the environmental event has anything at stake for him or her, and secondary appraisal, in which the individual evaluate if anything can be done to prevent harm or control the stimuli events (35, 37, 38). Lazarus (6) and Folkman et al. (37) suggested that the results of appraisal influence individuals’ psychological well-being and emotional responses.

In the early stage of the COVID-19 pandemic in China, COVID-19 brought new stimuli to the college students, such as concerns about one’s own or family’s physical health, freedom of social activities restricted by quarantine, fear of infection from the virus, insufficient information, and inadequate supplies (39, 40). When they were confronted with these stimuli from a COVID-19 outbreak, they would assess their ability to overcome or prevent the COVID-19 threat on their own or with government guidance. This appraisal results in their perceived threat avoidability of COVID-19 (coping appraisal) in this study. According to cognitive appraisal theory (37), the perceived threat avoidability of COVID-19 would affect their psychological well-being. If students believe that they can effectively prevent COVID-19 by taking some COVID-19 precautions, which means they have a high level of perceived threat avoidability of COVID-19. In that case, their psychic anxiety symptoms will be alleviated. Recent studies have also confirmed that perceived controllability, in turn, alleviates students’ anxiety levels (24, 28). Based on this discussion, we propose the following hypothesis:

H1: Perceived threat avoidability of COVID-19 negatively correlates with college students’ psychic anxiety.

### Mediating Effect of COVID-19-Specific Wishful Thinking

Coping is another core concept of cognitive appraisal theory, defined as the person’s cognitive and behavioral efforts to manage specific external and/or internal demands that are appraised as taxing exceeding the person’s resources (23). The processes of coping are divided into two types: EFC, which refers to pacifying or controlling the emotion aroused by the stressful situation, or to dismiss the emotional discomforts, and PFC, which refers to doing something to change for the better the problem causing the distress (35, 38). Folkman et al. (26) indicated that coping is highly correlated with cognitive appraisal and that different types of coping styles can have different effects on psychological symptoms (37). In other words, coping strategies play a mediating role between cognitive appraisal and psychological well-being.

Wishful thinking is a form of EFC, in which the individual avoids the effects of an environmental event by fantasizing or hoping that the situation will disappear or end suddenly, which is an escape-avoidance type of coping (26) and is a negative non-adaptive coping strategy (31). In the context of the COVID-19 pandemic, when people perceive that the threat is avoidable and the harm can be avoided through some measures, they will reduce the use of EFC such as wishful thinking (36). At the same time, previous studies have shown that wishful thinking negatively affects individuals’ mental health (41), is predictive of negative emotions (34), and increases the levels of anxiety (42, 43). Based on this, we propose the following hypothesis:

H2: COVID-19-specific wishful thinking mediates the relationship between perceived threat avoidability of COVID-19 and psychic anxiety.

### Mediating Effect of COVID-19-Specific Protective Behaviors

In the context of the COVID-19 pandemic, various protective behaviors (e.g., wearing protective devices outside the home, reducing exposure to others, washing hands, etc.) in response to the pandemic’s prevention and control can be considered a form of PFC (44). If people are aware that they can prevent infection or reduce harm from COVID-19 by taking specific COVID-19 coping measures (high perceived threat avoidability). In that case, they will tend to actively adopt COVID-19 protective behaviors to protect their health and lives (24). According to cognitive appraisal theory (37), COVID-19-specific protective behaviors, as a PFC strategy, are not only influenced by perceived threat avoidability but also alleviate anxiety symptoms. Recent studies with Turkish health care workers (45) and Chinese university students (19) have shown a negative relationship between PFC and anxiety in response to the COVID-19 outbreak. Thus, this paper proposes the following hypothesis for the mediating effect of COVID-19-specific protective behaviors:

H3: COVID-19-specific protective behavior mediates the relationship between perceived threat avoidability of COVID-19 and psychic anxiety.

### The Relationship Between COVID-19-Specific Wishful Thinking and COVID-19-Specific Protective Behaviors

Folkman and Lazarus (35) suggested a correlation between EFC and PFC strategies and that different types of EFC strategies have different effects on PFC strategies. Wishful thinking, a form of EFC strategy, refers to an individual’s effort to cognitively escape from or avoid a situation by simply fantasying or hoping the situation will go away or somehow be over (41). It will lead to individuals’ misperceptions of the threat. Then people are not motivated to take PFC strategies, because they are not sufficiently concerned about the situation and are less likely to take protective measures (36). Research in the information technology threat domain has shown that inward EFC strategies, including wishful thinking, has a negative effect on PFC strategies. In the context of the COVID-19 pandemic, COVID-19-specific wishful thinking could have a negative effect on COVID-19-specific protective behaviors. Thus, this paper proposes the following hypothesis.

H4: COVID-19-specific wishful thinking is negatively associated with COVID-19-specific protective behaviors.

## Materials and Methods

### Sample

This research used a random sampling method and the data was collected *via* an online questionnaire survey on the Wenjuanxing^1^ survey platform. The respondents are college students from 10 universities located in Anhui, central China. This research was conducted from 6 February 2020 to 25 February 2020 to obtain college students’ data in the early stage of the COVID-19 pandemic. These ten universities were selected out of 115 universities in Anhui province using the random number table method. We contacted the counseling agencies of these 10 universities and asked them to randomly select 2–3 counselors. Then we distributed the hyperlink and quick response (QR) code of the questionnaire to these selected counselors of each college, who further distributed the hyperlink and QR code to the students of their respective colleges. The questionnaire could be accessed and completed by participants *via* computer, mobile phone, or pad. The setting function of the Wenjuanxing survey platform was requested that one questionnaire could only be completed once for each IP address to ensure the validity of the questionnaire. A total of 3,088 questionnaires were collected in this research. After eliminating 166 invalid questionnaires with short response time, missing values, and consistency of question items, 2,922 valid questionnaires were retained. This research was approved by the Research Ethics Committee of School of Business at Anhui University of Technology (SB-AHUT-REC-2020-02-HS01). All participants gave their informed consent for inclusion prior to the survey.

Of the 2922 participants, 40.3% (*n* = 1,179) were male, 59.7% (*n* = 1,743) were female, and the mean age was 19.91 (*SD* = 1.48). The percentage of students in economics and management was 49.7% (*n* = 1,451), 27. 5% (*n* = 803) in science and engineering, 10.4% (*n* = 305) in humanities, 10.2% (*n* = 298) in arts, and 2.2% (*n* = 65) in other categories. The percentages of students with a health status of “very poor” was 0.1% (*n* = 2), of “poor” was 1.0% (*n* = 28), of “average” was 20.4% (*n* = 596), of “good” was 47.2% (*n* = 1,380), and of “very good” was 31.3% (*n* = 916). The percentage of students living in the hospitals and unified quarantine was 0.3% (*n* = 9), 14.9% (*n* = 436) in high-risk areas and unified quarantine, 78.4% (*n* = 2,291) in high-risk areas and self-quarantine, 4.7% (*n* = 137) in medium-risk areas and self-quarantine, 1.7% (*n* = 49) in low-risk areas. The participants were distributed in Anhui, Jiangsu, Zhejiang, Shandong, Hunan, Hubei, Henan, Hebei, Guangdong, Gansu, Inner Mongolia, Xinjiang and other provinces. The demographic characteristics of the study sample are shown in Table 1.

**TABLE 1:** Baseline/socio-demographic characteristics of the sample (*N* = 2,922).

Category	Frequency	Percentage
**Sex**		
Male	1,179	40.3
Female	1,743	59.7
**Age**	**Mean = 19.91; *SD* = 1.48**	
**Speciality**		
Economics and management	1,451	49.7
Science and engineering	803	27.5
Humanities	305	10.4
Arts	298	10.2
Other	65	2.2
**Health status**		
Very poor	2	0.1
Poor	28	1.0
Average	596	20.4
Good	1,380	47.2
Very good	916	31.3
**Risk level of living area**		
Hospitals and unified quarantine	9	0.3
High-risk areas and unified quarantine	436	14.9
High-risk areas and self-quarantine	2,291	78.4
Medium-risk areas and self-quarantine	137	4.7
Low-risk areas	49	1.7

*SD, standard deviation.*

### Measurements

We adapted and revised several scales or multi-items to measure the perceived threat avoidability of COVID-19, the COVID-19-specific wishful thinking, and the COVID-19-specific protective behaviors. Two bilingual experts (Chinese and English) translated the original scales from English to Chinese in parallel, and two other bilingual scholars conducted a back-translation. Next, proper adjustments were made accordingly after discussing and identifying inconsistent contents between the original and back-translated versions. Finally, we slightly adjusted the items to fit the COVID-19 pandemic in the Chinese context. Psychic anxiety was measured using a Chinese revision of The Hamilton Anxiety Scale (HAMA) widely used in China. Before conducting hypotheses testing, we examined all scales for reliability, convergent validity and discriminant validity, and the results indicated that they all had good psychometric properties in the present study.

#### Perceived Threat Avoidability of COVID-19

In this research, the perceived threat avoidability of COVID-19 was measured using three items which were revised from the Perceived Avoidability Scale developed by Liang et al. (36) to better reflect the context of the COVID-19. The following are the three items: “The threat posed by COVID-19 can be prevented,” “I can protect myself from the COVID-19 threat,” and “Overall, I think the COVID-19 threat is manageable.” All items were 7-point Likert scaled (1 = strongly disagree, 7 = strongly agree), with higher scores indicating higher levels of perceived threat avoidability of COVID-19. The Cronbach’s α of this scale was 0.831.

#### COVID-19-Specific Wishful Thinking

COVID-19-specific wishful thinking was measured using four items which were revised from the Wishful Thinking Scale developed by Liang et al. (36) to reflect the context of the COVID-19 better. The following are the four items: “I fantasized that COVID-19 would go away or somehow be over with,” “I fantasized that I would somehow come across a magical solution for it,” “I fantasized that all of a sudden COVID-19 disappears by itself,” and “I fantasized that everything turns out just fine as if nothing happened.” All items were 7-point Likert scaled (1 = strongly disagree, 7 = strongly agree), with higher scores indicating higher levels of EFC with wishful thinking. The Cronbach’s α of this scale was 0.874.

#### COVID-19-Specific Protective Behaviors

Due to the lack of a COVID-19-specific Protective Behaviors Scale, we developed a 5-item scale based on the safety protective measures against COVID-19 recommended by the WHO (46) and the Chinese Center for Disease Control and Prevention. The following are the five items: “Wearing protective equipment when going out,” “Reducing contact with others,” “Enhancing personal hygiene,” “Enhancing family hygiene,” and “Cleaning yourself when you come home from outside.” All items were 5-point Likert scaled (1 = never and 5 = always), with higher scores indicating higher levels of COVID-19-specific protective behaviors. The Cronbach’s α of this scale was 0.878.

#### Psychic Anxiety

The HAMA is widely used to assess anxiety levels around the world, and the Chinese version of the HAMA used in the present study has been widely used in the Chinese population, and its psychometric properties have been effectively validated (47). The HAMA is one of the first scales commonly used in psychiatric clinics and contains 14 items (48). The HAMA classifies anxiety factors into two categories: somatic and psychic anxiety. We selected seven items on psychic anxiety, namely the Hamilton Psychogenic Anxiety Scale (HAMA-PSY). All items were 5-point Likert scaled (1 = never and 5 = always), which contained the following seven items: “I feel worried, concerned, and feel that the worst thing is going to happen,” “I feel uneasy, nervous, and cannot relax,” “I am afraid of being alone, in a car, going out and in crowds,” “I have difficulty sleeping, wake up easily, dream a lot, wake up tired,” “I have difficulty concentrating, poor memory,” “I lose interest in past hobbies, depression, early awakening,” and “I am nervous, apprehensive, shaking hands, frowning, stiff expressions, swallowing, fast heartbeat, fast breathing, fluttering eyelids, easy sweating when communicating with others.” The Cronbach’s α of this scale was 0.890.

### Analysis Strategy

Data analysis was performed by the statistical package for Social Science (IBM-SPSS) v26.0 and Mplus v8.3. Firstly, to test whether there was a common method bias problem for the research dataset, Harman’s one-factor test was conducted with IBM-SPSS (v26.0). If the variance explained by the first principal component was less than 50% of the total variance, which indicates a low probability of common method bias (49). Secondly, confirmatory factor analysis (CFA) was conducted with Mplus (v8.3) to further validate the results of Harman’s one-factor test, comparing the Chi-square (χ^2^) and degree of freedom (*df*) of the four-factor model and the one-way model. If the χ^2^ and *df* of the four-factor model were significantly lower than the one-way model, which further indicated that the common method bias problem of the research was not significant (50). Scale reliability, validity, and correlation analysis were conducted with IBM-SPSS (v26.0) before conducting model hypothesis testing. The Cronbach’s alpha is used to evaluate the reliability of the scale. The Cronbach’s alpha coefficient is greater than the cutoff value of 0.70, indicating that the scale has good reliability (51). Then, the hypotheses proposed in this study were tested by Mplus (v8.3). Several commonly used fit indices were used to evaluate the model, including χ^2^(*df*), Comparative Fit Index (CFI), Tucker Lewis Index (TLI), Standardized Root Mean Square Residual (SRMR), and Root Mean Square Error of Approximation (RMSEA). Since χ^2^ test is highly affected by sample size (52), when the sample is large, it can lead to an inflated χ^2^ statistic (53). Therefore, the model fitted better when CFI and TLI > 0.95, SRMR < 0.08 and RMSEA < 0.08 (54). Finally, the bias-corrected non-parametric percentile Bootstrap method was used to test the mediating effects by Mplus (v8.3) with 95% confidence interval and 5,000 iterations. If the 95% confidence interval does not contain 0, the mediating effect is significant (55). In the model analysis, perceived threat avoidability of COVID-19 was analyzed as the independent variable, psychic anxiety was analyzed as the dependent variable, and COVID-19-specific wishful thinking and COVID-19-specific protective behaviors were analyzed as mediating variables. In terms of control variables considered, sex, age, health status, and risk level of living area were initially considered in this study based on previous studies (56–58), and then the control variables to be included in the model calculation will be determined based on the results of correlation between these variables and the dependent variable.

## Results

### Reliability, Validity Analysis, and Correlation Analysis

The Cronbach’s alpha is used to evaluate the reliability of the scale (51). As shown in Table 2, each scale’s Cronbach’s alpha coefficient in this study was greater than 0.7, showing that the scales have reliability (51). Standardized factor loadings and average variance extracted (AVE) values were used to evaluate convergent validity. Table 2 shows that all observed variables had standardized factor loadings larger than 0.5 (59), the AVE values of each factor were greater than 0.5 (60), and the composite reliability (CR) values ranged from 0.834 to 0.879, all of which were greater than 0.8 (51). These findings suggested that the scales employed in this study had good convergent validity. The discriminant validity of the scale was evaluated using the square root of AVE and the correlation coefficient between factors. Table 3 shows the variables’ mean, standard deviation, and correlation coefficients for all variables as well as the square root of AVE for four latent variables. According to the results provided in Table 3, the square root of AVE (bold values on the diagonal of Table 3) is greater than the correlation coefficients between the variables, indicating that the scales have good discriminant validity (61). Meanwhile, perceived threat avoidability of COVID-19 was negatively correlated with COVID-19-specific wishful thinking (*r* = −0.040, *p* < 0.05) and psychic anxiety (*r* = −0.226, *p* < 0.001), but positively correctly with COVID-19-specific protective behaviors (*r* = 0.192, *p* < 0.001). COVID-19-specific wishful thinking was negatively correlated with COVID-specific protective behaviors (*r* = −0.109, *p* < 0.001), and was positively correlated with psychic anxiety (*r* = 0.130, *p* < 0.001). Meanwhile, COVID-specific protective behaviors were negatively correlated with psychic anxiety (*r* = −0.193, *p* < −0.001). For the control variables, health status (*r* = −0.227, *p* < 0.001) and age (*r* = 0.128, *p* < 0.001) were significantly correlated with psychic anxiety, while sex (*r* = 0.034, *p* = 0.068) and risk of living area (*r* = 0.004, *p* = 0.830) were not significantly correlated with psychic anxiety. Therefore, these two variables were included as control variables in the subsequent structural equation modeling and mediation tests. The correlation coefficient results provide preliminary support for the hypotheses.

**TABLE 2:** Results of reliability and validity.

Factor	Factor loadings	Cronbach’s alpha	AVE	CR
COVID-19 PTA	0.727∼0.855	0.831	0.627	0.834
COVID-19 WT	0.700∼0.877	0.874	0.645	0.878
COVID-19 PB	0.621∼0.864	0.878	0.592	0.877
PA	0.508∼0.858	0.890	0.518	0.879

*AVE, average variance extracted values; CR, composite reliability values; COVID-19 PTA, Perceived threat avoidability of COVID-19; COVID-19 WT, COVID-19-specific wishful thinking; COVID-19 PB, COVID-19-specific protective behaviors; PA, psychic anxiety.*

**TABLE 3:** Mean, SD, correlation coefficients, and square root of average variance extracted values.

	Mean	*SD*	1	2	3	4	5	6	7
1. COVID-19 PTA	5.610	0.872	**0.792**						
2. COVID-19 WT	3.348	1.402	−0.040*	**0.803**					
3. COVID-19 PB	4.534	0.567	0.192***	−0.109***	**0.769**				
4. PA	2.028	0.670	−0.226***	0.130***	−0.193***	**0.720**			
5. Sex	–	–	−0.099***	0.036	0.136***	0.034			
6. Health status	4.090	0.745	0.197***	–0.035	0.181***	−0.227***	−0.084***		
7. Risk level of living area	1.990	0.699	–0.011	–0.036	0.027	0.004	0.043*	−0.054**	
8. Age	19.910	1.475	−0.060**	0.048**	−0.048**	0.128***	0.028	−0.059**	-0.051**

*p < 0.05; **p < 0.01; ***p < 0.001.

*SD, standard deviation; COVID-19 PTA, Perceived threat avoidability of COVID-19; COVID-19 WT, COVID-19-specific wishful thinking; COVID-19 PB, COVID-19-specific protective behaviors; PA, psychic anxiety.*

*Bold values on the diagonal are the square root of average variance extracted (AVE) values.*

### Common Method Bias

Considering that the self-reported data collected in this research is subjective in nature, the results may be influenced by common method bias (CMB) (62). To test whether CMB exists in the dataset of this research, this paper used Harman’s one-factor test to conduct an unrotated exploratory factor analysis on all scale question items. The first principal component explained 27.228% of the variance, which was lower than 50% of the total variance, indicating that the likelihood of the existence of CMB in the data was low. Considering the problems with the Harman’s one-factor test (63), this study used confirmatory factor analysis (CFA) to further test for CMB. The CFA results displayed in Table 4 show that fitting results of the four-factor model [χ^2^_(_*_*df*_*_)_ = 637.632 (141), CFI = 0.984 TLI = 0.980, SRMR = 0.031, RMSEA = 0.035] was obviously better than the single-factor model [χ^2^_(_*_*df*_*_)_ = 16343.193 (147), CFI = 0.468, TLI = 0.381, SRMR = 0.194, RMSEA = 0.194] and Δχ^2^ (Δ*df*) = 15705.561 (6), *p* < 0.001, indicating that there was no significant CMB in the data set of this research.

**TABLE 4:** Fit indices of the factor models.

	χ ^2^(*df*)	RMSEA	SRMR	CFI	TLI	Δχ ^2^(Δ *df)*
Four-factor model	637.632 (141)	0.035	0.031	0.984	0.980	–
Three-factor model	7384.090 (144)	0.131	0.138	0.762	0.718	6,746.458 (3)***
Two-factor model	9992.695 (146)	0.152	0.137	0. 676	0. 621	2,608.605 (2)***
Single-factor model	16343.193 (147)	0.194	0.194	0.468	0.381	6350.498 (1)***

***p < 0.001.

*RMSEA, Root Mean Square Error of Approximation; SRMR: Standardized Root Mean Square Residual; CFI: Comparative Fit Index; TLI: Tucker Lewis index; **Δ** χ^2^: Chi-square value increment; **Δ** df: degree of freedom increment.*

### Hypothesis Testing

**FIGURE 1:**
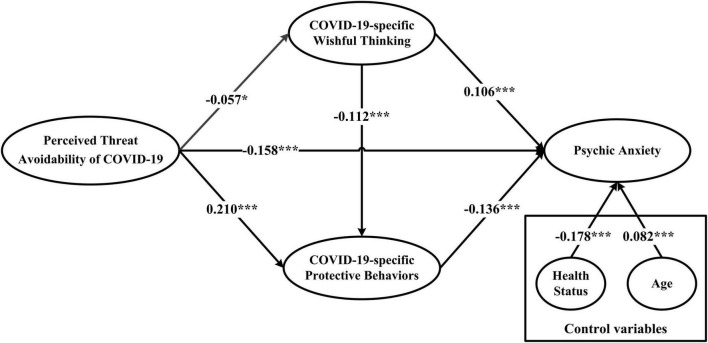
Structural model testing results, Path coefficients are standardized (*N* = 2,922; *p < 0.05, ***p < 0.001). χ^2^/*df* = 5.213, CFI = 0.976, TLI = 0.971, SRMR = 0.046, RMSEA = 0.038. Two control variables (health status and age) were included in the model.

After incorporating demographic variables (age and health status) as control variables into the structural equation model, the model fit indices (χ^2^/*df* = 5.213, CFI = 0.976, TLI = 0.971, SRMR = 0.046, RMSEA = 0.038) indicated that the hypothesized model fit was well. The results are presented in Figure 1. Perceived threat avoidability of COVID-19 was negatively related to psychic anxiety (β = −0.158, *p* < 0.001), thus supporting H1. Perceived threat avoidability of COVID-19 negatively correlated with COVID-19-specific wishful thinking (β = −0.057, *p* < 0.05), which, in turn, positively related to psychic anxiety (β = 0.106, *p* < 0.001), thus providing preliminary evidence for H2. Perceived threat avoidability of COVID-19 positively correlated with COVID-19-specific protective behaviors (β = 0.210, *p* < 0.001), which, in turn, negatively related to psychic anxiety (β = −0.136, *p* < 0.001), thus providing preliminary evidence for H3. Finally, COVID-19-specific wishful thinking had a negative relation to COVID-19-specific protective behaviors (β = −0.112, *p* < 0.001), thus supporting H4.

### Mediating Effect Test

The bias-corrected non-parametric percentile bootstrap method was used to examine the mediating effects of COVID-19-specific wishful thinking and COVID-19-specific protective behaviors in the relationship between perceived threat avoidability of COVID-19 and psychic anxiety by Mplus (v8.3), and the results of the analysis are presented in Table 5. The findings suggest the indirect effect of perceived threat avoidability of COVID-19 on psychic anxiety through COVID-19-specific wishful thinking was significant (β = −0.006, 95% CI: [−0.012, −0.001], not including 0), thus supporting H2. As expected in H3, the indirect effect of COVID-19-specific protective behaviors was significant (β = −0.029, 95% CI: [−0.043, −0.018], not including 0).

**TABLE 5:** Results of mediating effects.

	Estimate	S.E.	*p*-value	95% CI	
				Lower	Upper
**COVID-19 PTA → PA**					
Total	–0.193	0.023	0.000	–0.239	–0.147
Direct	–0.158	0.024	0.000	–0.203	–0.111
Total indirect	–0.035	0.007	0.000	–0.050	–0.024
**Indirect**					
COVID-19 PTA → COVID-19 WT → PA	–0.006	0.003	0.029	–0.012	–0.001
COVID-19 PTA → COVID-19 PB → PA	–0.029	0.006	0.000	–0.043	–0.018

*S.E., standard error; CI, confidence interval; COVID-19 PTA, Perceived threat avoidability of COVID-19; COVID-19 WT, COVID-19-specific wishful thinking; COVID-19 PB, COVID-19-specific protective behaviors; PA, psychic anxiety. The bias-corrected non-parametric percentile Bootstrap method, with 95% confidence interval and 5000 iterations, was used to test the mediating effects by Mplus (v8.3). The COVID-19 PTA was analyzed as the independent variable, PA was analyzed as the dependent variable, and COVID-19 WT and COVID-19 PB were analyzed as mediating variables. Control variables included health status and age.*

## Discussion

Grounded on cognitive appraisal theory, the current study examined the impact of perceived threat avoidability of COVID-19 on psychic anxiety among college students, the mediating role of COVID-19-specific wishful thinking and protective behaviors in the early stage of the COVID-19 pandemic, and the effect of COVID-19-specific wishful thinking on COVID-19-specific protective behaviors. The findings show that perceived threat avoidability is related to college students’ psychic anxiety. College students with low threat avoidability experience high psychic anxiety, further supporting prior findings focusing on the general samples in China who came from various provinces, ranging in age from 17 to 90 years old, with various statuses of education and health (24). As we expected, COVID-19-specific wishful thinking, a negative EFC strategy, plays a mediating role in the relationship between perceived threat avoidability and psychic anxiety. Consistent with prior findings, we confirm that when individuals perceive a high level of threat avoidability, they will reduce wishful thinking (36) and experience a low level of anxiety symptoms (43, 64). Furthermore, our results support the mediating effect of COVID-19-specific protective behaviors, a positive PFC strategy, in the relationship between perceived threat avoidability and psychic anxiety. These findings confirm the argument that perceived threat avoidability influences individuals’ positive PFC behaviors (36), which, in turn, reduces their anxiety symptoms (19, 65, 66). Finally, our results found that COVID-19-specific wishful thinking has a negative effect on COVID-19-specific protective behaviors. In addition, the results also showed that health status and age among the control variables were significantly associated with psychic anxiety. A possible reason for the higher levels of mental anxiety among college students in poorer health is that students in poorer health are more likely to suffer from health impairment due to COVID-19 (57) and therefore feel higher levels of anxiety, and a possible reason for the higher levels of mental anxiety among older students is that seniors now face considerable uncertainty regarding their educational and economic futures (67) and therefore feel higher levels of anxiety.

This study makes several contributions to the literature on the psychic anxiety effect of COVID-19 and the cognitive appraisal theory. First, drawing upon cognitive appraisal theory (6, 26), the paper contributes to our understanding of COVID-19 induced psychic anxiety by identifying secondary appraisal, i.e., perceived threat avoidability in this study, which has been underestimated. Previous studies have well documented the influence of primary appraisal on anxiety from the perspective of perceived threat susceptibility and severity of COVID-19 (27, 68), but few researchers have examined the influence of perceived threat avoidability on psychic anxiety. This omission could seriously limit our understanding of the different levels of psychic anxiety among college students. The current study focuses on the neglected important role of college students’ perception of threat avoidability in predicting psychic anxiety in the early stage of the COVID-19 pandemic, and thus greatly extends COVID-19 studies on threat and mental health.

Second, we reveal the roles of two different forms of coping strategies in mediating the relationship between perceived threat avoidability and psychic anxiety based on the cognitive appraisal theory. The studies that have been conducted on the mediating mechanisms of coping strategies in COVID-19 threat and anxiety may focus on external ones, such as quarantine strategies (29), and lack the exploration of internal cognitive coping strategies. Whereas in studies of internal cognitive coping strategies, although existing studies have investigated the direct effects of coping strategies on anxiety (19, 20), little is known about the antecedents of EFC and PFC coping strategies and whether these two types of coping strategies mediate the relationship between perceived threat avoidability and psychic anxiety. This study firstly focused on important negative EFC coping strategies (i.e., COVID-19-specific wishful thinking) and positive PFC coping strategy (i.e., COVID-19-specific protective behavior) toward COVID-19 threat, and revealed how perceived threat avoidability reduces psychic anxiety *via* decreasing wishful thinking and increasing protective behaviors. By doing so, we not only support previous findings of the effect of perceived threat avoidability on wishful thinking and protective behaviors (36), also open the “black box” between COVID-19-specific threat avoidability and psychic anxiety.

Third, this study complements and extends cognitive appraisal theory by theorizing and validating the relationship between two specific strategies (EFC and PFC). Although Folkman and Lazarus (35) argued that some forms of EFC strategies might impede PFC strategies, a recent study by Liang et al. (36) further demonstrated such effect of inward EFC on PFC behaviors in the context of information technology threat, little study has updated this effect in the context of human life and health threat. As a response, this study draws attention to COVID-19-specific wishful thinking (a specific form of inward EFC), and demonstrates that COVID-19-specific wishful thinking negatively correlates with COVID-19-specific protective behaviors. Advancing a step beyond previous studies examining the independent role of coping strategies (19, 20), the present study improves the understanding of the joint role of different coping strategies in COVID-19-related psychic anxiety. This finding thus contributes to cognitive appraisal literature by providing evidence to the argument of Folkman and Lazarus (35) and supporting the prior finding of Liang et al. (36) in a different context.

Our study has several practical implications for mental health management practice. First, the findings of this study suggest that college students’ perceived threat avoidability is negatively associated with their psychic anxiety. It means that the level of psychic anxiety among college students can be mitigated by increasing their perceived threat avoidability when facing a serious life and health threat from public health emergencies such as the COVID-19 pandemic. For example, the governments are recommended to announce information about COVID-19 immediately on official websites, clarify social rumors, and invite reputable experts to popularize knowledge of COVID-19. These initiatives can educate college students to recognize the threat controllability of COVID-19 scientifically and accurately, thus increasing their perceived threat avoidability (24).

Second, our findings suggest that COVID-19-specific protective behaviors contribute to low psychic anxiety among college students. It is consistent with the established beneficial effects of positive protective behaviors in the COVID-19 pandemic (19, 69). Therefore, prevention policy-makers and college administrators should develop scientific and rigorous safety measures, such as strict social isolation, regular window ventilation, wearing masks, and washing hands correctly when going out, and guide college students, to abide by these safety measures. In this case, college students’ confidence could be increased in avoiding infection with and fighting off COVID-19.

Third, our findings also indicate that COVID-19-specific wishful thinking increases psychic anxiety directly and indirectly by impeding COVID-19-specific protective behaviors, which offer a new direction to the practice of mental health management. Therefore, we argue that it is equally important to educate college students (and the general public) to understand the potentially harmful effect of wishful thinking (34, 41) and give up this negative EFC strategy. For example, mental health education or counseling institutions could design psychological coaching programs to help students be aware of how wishful thinking generates and affects their psychic anxiety.

Some methodological limitations in this study should be further noted. First, this study collected college students’ self-reported data, which may be affected by social desirability limitations inherent in most research (70). Future research can minimize this limitation by taking precautions to combat socially desirable responses recommended by Mick (71). Second, we used cross-sectional data to test the hypothesized model, implying the inability to draw causal conclusions (72, 73). Future studies can reexamine the causal connections by incorporating the experimental or longitudinal design. A third methodological limitation is related to the representativeness of the present sample. The current survey was completed by college students from 10 universities in Anhui province. Therefore, potential selection biases might have influenced the generalization of our findings. Hence, more studies are recommended to replicate the present findings with more representative samples from more universities in other provinces in China, which may bolster the relevance of such findings to a broader audience.

## Data Availability Statement

The raw data supporting the conclusions of this article will be made available by the authors, without undue reservation.

## Ethics Statement

The current study was approved by the Ethics Committee entitled “Institutional Review Board of the School of Business, Anhui University of Technology.” The patients/participants provided their written informed consent to participate in this study.

## Author Contributions

JW, YZ, and SX co-drafted and wrote the manuscript. YZ, JW, and WZ revised the manuscript and finally approved the version to be published. YZ and SX analyzed and interpreted the data. SG provided feedback to improve the manuscript. JW supervised the data gather process. All authors contributed to the article and approved the submitted version.

## Conflict of Interest

The authors declare that the research was conducted in the absence of any commercial or financial relationships that could be construed as a potential conflict of interest.

## Publisher’s Note

All claims expressed in this article are solely those of the authors and do not necessarily represent those of their affiliated organizations, or those of the publisher, the editors and the reviewers. Any product that may be evaluated in this article, or claim that may be made by its manufacturer, is not guaranteed or endorsed by the publisher.

## Footnotes

1
www.wjx.cn
